# Progression of duodenal neoplasia to advanced adenoma in patients with familial adenomatous polyposis

**DOI:** 10.1186/s13053-023-00264-2

**Published:** 2023-11-27

**Authors:** Hiroko Nakahira, Yoji Takeuchi, Yusaku Shimamoto, Shingo Ishiguro, Hiroshi Yunokizaki, Yasumasa Ezoe, Fumie Fujisawa, Ryu Ishihara, Tetsuji Takayama, Teruhiko Yoshida, Michihiro Mutoh, Hideki Ishikawa

**Affiliations:** 1 Department of Gastrointestinal Oncology, Osaka, Japan; 2 Department of Genetic Oncology, Division of Hereditary Tumors, Osaka International Cancer Institute, Osaka, Japan; 3 Department of Gastroenterology and Hepatology, Gunma University Graduate School of Medicine, 3-39-15, Showa-machi, 371-8511 Maebashi, Gunma Japan; 4 Pathology & Cytology Laboratories Japan, Tokyo, Japan; 5 Ishikawa Gastroenterology Clinic, Osaka, Japan; 6 Department of Gastroenterology and Oncology, Institute of Biomedical Sciences, Tokushima University Graduate School, Tokushima, Japan; 7 Department of Genetic Medicine and Services, National Cancer Center Hospital, Tokyo, Japan; 8 Department of Molecular-Targeting Cancer Prevention, Graduate School of Medical Science, Kyoto Prefectural University of Medicine, Kyoto, Japan

**Keywords:** Familial adenomatous polyposis, Non-ampullary duodenal adenoma, Advanced adenoma, Endoscopic surveillance, Progression

## Abstract

**Background:**

Patients with familial adenomatous polyposis (FAP) have a lifetime risk of developing duodenal adenomas approaching 100%, and the relative risk for duodenal cancer compared with the general population is high. We conducted a retrospective study to investigate the progression of non-ampullary duodenal adenomas (NADAs) and risk factors for advanced lesions in patients with FAP.

**Methods:**

Of 248 patients with 139 pedigrees at 2 institutes, we assessed 151 patients with 100 pedigrees with a pathogenic germline variant in the *adenomatous polyposis coli* gene, excluding mosaic variants. We evaluated the prevalence of NADAs in patients with FAP, the progression of these adenomas to advanced adenoma during the observation period, and the risk factors for the lifetime development of high-grade dysplasia (HGD), large (≥ 10 mm) duodenal adenomas, and Spiegelman stage IV.

**Results:**

During the median observation period of 7 years, the incidences of patients with NADAs, with more than 20 polyps, with polyps ≥ 10 mm, with HGD, and with stage IV at the last esophagogastroduodenoscopy were increased 1.6-fold, 1.7-fold, 5-fold, 22-fold, and 9-fold, respectively. Intramucosal cancer occurred in three patients (2%), but no patients developed invasive cancer during the observation period because we performed endoscopic intervention for advanced adenomas. Stage progression was observed in 71% of 113 patients. Stage IV was more common in women, patients with a history of colectomy, and those with a 3’ side mutation in their *adenomatous polyposis coli* gene.

**Conclusions:**

NADAs in patients with FAP frequently become exacerbated. Our findings suggest that patients with FAP who develop duodenal adenomas should be surveyed to prevent the development of duodenal cancer.

## Background

Familial adenomatous polyposis (FAP) is an autosomal dominant disorder caused by germline mutations in the *adenomatous polyposis coli* (*APC*) gene. It is characterized by multiple adenomas throughout the colon and rectum and results in colorectal cancer (CRC) in almost 100% of patients if left untreated.

FAP is associated with multiple lesions other than those in the large intestine, and the lifetime risk of developing duodenal adenomas approaches 100% [1–6]. Unlike adenomas in the large intestine, duodenal adenomas do not necessarily become cancerous. Nevertheless, the risk of duodenal cancer in patients with FAP is 250.0 to 330.8-fold higher than that of the general population [7, 8]. Therefore, patients with FAP constitute a high-risk group for duodenal cancer. The lifetime risk of developing duodenal cancer ranges from 3 to 5% [9–11] and is higher in other reports [1, 4, 12, 13]. Duodenal cancer is also the third leading cause of death in patients with FAP, accounting for the deaths of approximately 3% of these patients [14, 15]. Although half of duodenal cancer occur in the ampulla and periampullary region, we should consider ampullary and non-ampullary duodenal adenomas (NADAs) separately because ampullary adenoma rarely progresses, and when it does, it is slow [16]. Although a therapeutic strategy for ampullary adenoma has almost been established, no strategy for NADAs has been established.

CRC that occurs in patients with FAP can be managed by prophylactic total colectomy or intensive endoscopic intervention [17], leading to reduced mortality; thus, the prognosis of FAP has dramatically improved. With respect to duodenal cancer, the severity of NADAs is classified by Spigelman score. The risk of developing duodenal cancer is stratified according to its stage [18]. Because polyps larger than 10 mm and with high-grade dysplasia (HGD) are generally called “advanced adenoma” and are considered a precursor of cancer, we performed endoscopic interventions for these advanced adenomas. However, the extent to which NADAs worsen to advanced adenoma is unclear. Therefore, we proposed intensive downstaging polypectomy for all detected NADAs in our prospective study [19]. By examining the changes that occur in NADAs over time, we may more effectively stratify the risk of developing advanced duodenal adenomas. Therefore, we conducted a retrospective study to investigate the progression of NADAs and risk factors for advanced lesions in patients with FAP.

## Methods

### Patients

Patients with FAP who visited the Osaka International Cancer Institute and Ishikawa Gastrointestinal Clinic from January 1998 to September 2018 were retrospectively assessed for enrollment in this study. The diagnostic criteria for FAP in this study were (i) the presence of either ≥ 100 adenomas in the colon and rectum or 10 to 99 adenomas with a family history of FAP and (ii) a pathogenic germline variant in *APC*. Patients with mosaic variants in *APC* were excluded. Esophagogastroduodenoscopy (EGD) was generally recommended for all patients with FAP. Patients who had not undergone EGD at our institutions and patients with a history of duodenal surgery, such as gastric surgery or pancreatoduodenectomy, were not enrolled in this retrospective study because we could not ascertain the progression of the duodenal neoplasms in such patients. Most of the cases in this study were also analyzed in the study by Shimamoto et al. (Genotype-phenotype correlation for life-threatening complications in patients with familial adenomatous polyposis, accepted in *Cancer Science*).

### Extraction of clinical data and definition of measured variables

We collected clinical data from the patients’ medical and endoscopic records. The genetic information used in this study comprised *APC* pathogenic variant and pedigree data aggregated at the Medical Research Support Co., Ltd. (Osaka, Japan), a data center operated by academic doctors. The genetic information was aggregated and controlled at the center in accordance with strict guidelines. All patients received genetic counseling and provided informed consent for *APC* genetic testing.

The observation period was defined as the duration from the day on which the first EGD was performed to the day on which the last EGD was performed according to the patient’s medical record or until immediately before the treatment intervention, if any. We generally performed annual surveillance EGD and targeted biopsies of polyps. We considered treatment interventions for patients with an advanced duodenal adenoma, namely > 10 mm or HGD, defined as lesions with severe atypia according to the previous criteria, because advanced adenoma is generally considered a precursor of invasive cancer in colorectal polyp management, and we needed to prevent the development of duodenal cancer to secure the patients’ safety.

Based on the number of colorectal adenomas, severe FAP was defined as the inability to visualize a patient’s normal mucosa macroscopically because of the profusion of colorectal adenomas, and sparse FAP was considered typical FAP (which is not severe FAP). Attenuated FAP was defined as the presence of 10 to 99 colorectal adenomas. Patients were considered to have *Helicobacter pylori* infection when a positive result was obtained by a serological test, urea breath test, rapid urease test, or histological examination, and when they had a history of eradication therapy for *H. pylori*.

### Endoscopic system and settings

The endoscopic system consisted of a video processor (CV-260, CV-260SL, or CV-290; Olympus Co., Tokyo, Japan and VP-4450HD or VP-7000; Fujifilm Co., Tokyo, Japan) and a light source (CLV-260, CLV-260SL, or CLV-290; Olympus Co. and LL-4450, XL-4450, LL-7000, or BL-7000; Fujifilm Co.). A videoendoscope (GIF-H260Z, Q240Z, H290Z, PCF-Q260JI, or PCF-H290TL/I; Olympus Co. and EG-L590ZW, EG-L600ZW, or EGL600ZW7; Fujifilm Co.) was also used. Observations were performed by white light imaging and non-magnifying narrow-band imaging and/or with chromoendoscopy using indigo carmine with or without magnification.

### Histological examination

Lesions thought to be neoplastic or cancerous (well-demarcated lesions, large lesions, lesions with a depression at the center, and lesions with an irregular shape) were selected for biopsy. Biopsy tissue samples or endoscopic resection samples were collected and diagnosed. In patients who underwent intervention for duodenal neoplasms without prior biopsy, we collected information obtained by the following intervention. According to the Japanese classification of colorectal carcinoma, a histopathological examination was performed by two pathologists with expertise in gastrointestinal pathology. According to the Japanese classification, noninvasive cancer was evaluated as severe atypia by the Spigelman classification, which corresponds to Western standards. Because the grading of dysplasia according to the Vienna classification changed from mild/moderate/severe to low-grade/high-grade in 2000, our patients straddled the two eras. Our pathologists followed the mild/moderate/severe grading classification for a while, and then we adopted the original Spigelman stage (SS), using mild/moderate/severe in the analysis. We thus refer to severe atypia as “HGD” in this study. The most severe histopathological diagnosis during the observation period was adopted as the final histopathological diagnosis.

### Spigelman classification

When NADAs were found, they were scored using the Spigelman classification to indicate the severity of duodenal polyposis [18]. The Spigelman classification includes the number of polyps, maximum diameter, tissue structure, and degree of atypia. Stages 0 to IV were determined by the total score obtained using the above-mentioned criteria. When a detected duodenal polyp was not histopathologically diagnosed as an adenoma, it was not considered to be a NADA.

### Statistical analysis

We evaluated the prevalence of NADAs in patients with FAP, the progression of these adenomas during the observation period, the risk factors for the lifetime development of advanced adenoma, and SS IV until the end of the observation period.

The prevalence of NADAs in patients with FAP was indicated by the number and percentage of patients with NADAs. The progression of NADAs during the observation period was assessed with the SS, which includes the number of adenomas, size of adenomas, and development of severe dysplasia. Finally, we evaluated the lifetime risk of developing an advanced duodenal adenoma, and SS IV, which were reported to be risk factors for developing duodenal cancer [18, 20].

For the analysis of SS progression, patients for whom the observation period was < 5 years without SS progression were excluded because the observation period was considered too short. We then examined the progression of patients with SS 0 to III at the first EGD because there was no room for progression for patients with stage IV at the first EGD. We also examined the progression of patients with SS 0 and I at the first EGD, who were recommended to undergo follow-up at 5-year intervals according to the guidelines. Furthermore, we examined the occurrence of HGD according to the SS at the first EGD.

HGD, a tumor size ≥ 10 mm, the surveillance period, a history of colon cancer, age, the SS at the first EGD, classic FAP, and the location of several mutations in *APC* were evaluated as risk factors for disease progression because they were relevant to the severity of duodenal polyposis and reported to be risk factors for duodenal cancer [2–6, 21–23]. We also investigated whether *APC* mutations at codons 1250 to 1464, which are common in patients with severe FAP, were associated with the incidence and severity of NADAs [23]. Because we used only the protein truncation test for mutation detection in *APC* in patients whose first endoscopy procedure was performed before 2009, we could not obtain detailed information on the gene mutation site in some cases. We excluded those cases from the analysis of risk factors for disease progression.

Statistical analyses were performed using R software version 3.3.0 (R Foundation for Statistical Computing, Vienna, Austria; http://cran.r-project.org/). The data were analyzed using the χ^2^ test and Kruskal–Wallis test. Statistical significance was set at P < 0.05.

## Results

### Patient characteristics at first endoscopy

**Fig. 1:**
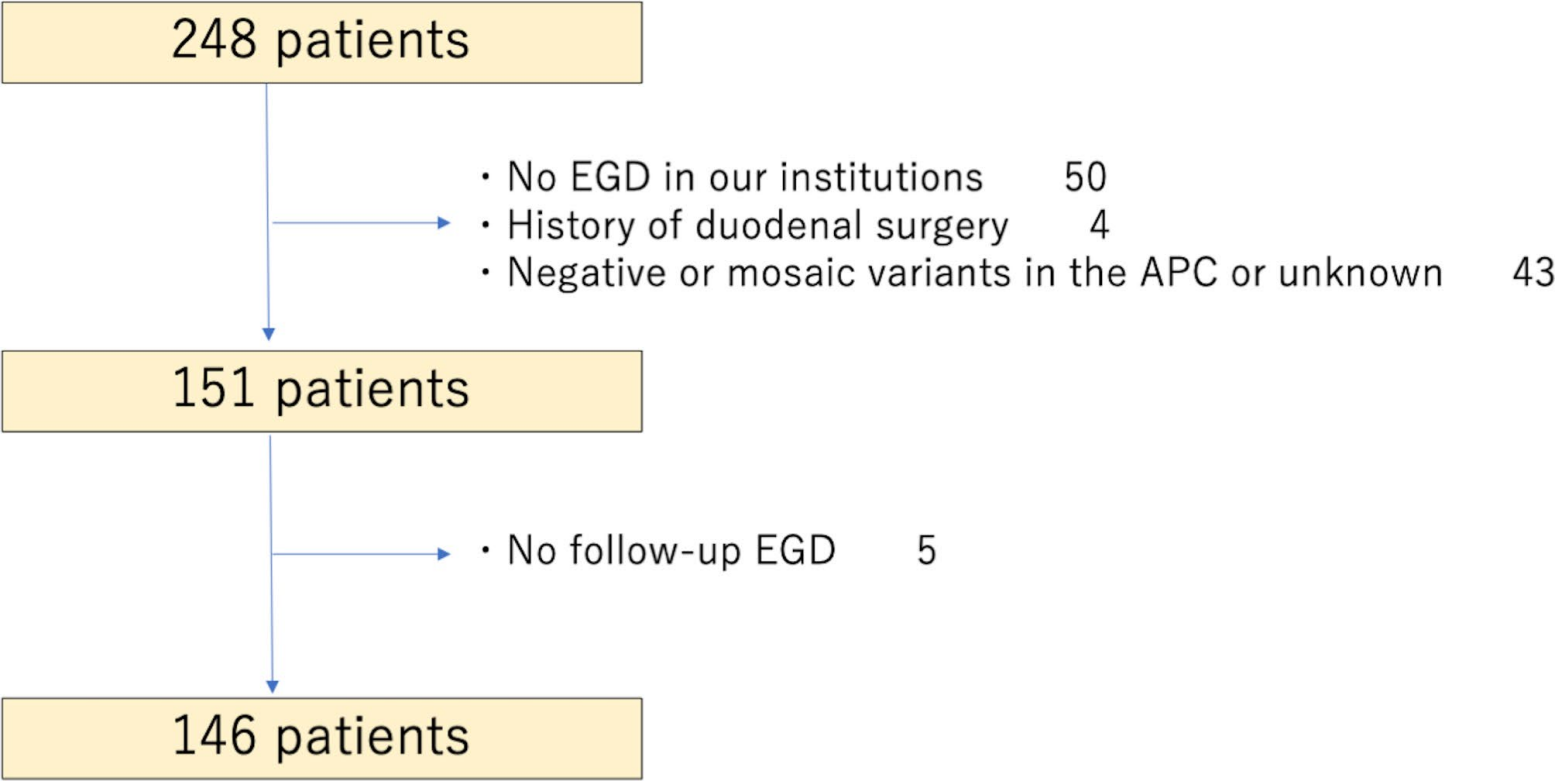
Flowchart of patient selection. EGD, esophagogastroduodenoscopy; *APC*, adenomatous polyposis coli gene

Among 248 patients from 139 pedigrees diagnosed with FAP from January 1998 to September 2019 at our institutes, 50 patients who had not undergone EGD at our institutes and 4 patients with a history of duodenal surgery were excluded. *APC* genetic testing was performed for 90% of the remaining 194 patients from 110 pedigrees, among whom 151 patients from 100 pedigrees were mutation-positive (Fig. 1). Table 1 shows the patients’ background characteristics. Female patients were predominant (57%), most patients had a family history of FAP, one-third had a history of colectomy, and a few (7%) had a history of CRC. An ampullary adenoma was found in 22 patients (15%), including 1 patient with a history of papillectomy.

**Table 1:** Patients’ baseline characteristics

Characteristics	n = 151
Age at first endoscopy, years	30 (16–76)
Sex, male	65 (43)
Body mass index, kg/m²	21.0 (14.7–37.2)
Alcohol intake	
Past/current drinker Never-drinker	74 (49) 77 (51)
Smoking	
Past/current smoker Never-smoker	36 (24) 115 (76)
Family history of FAP	
Positive Negative Unknown	130 (86) 8 (5) 13 (9)
*Helicobacter pylori* status	
Positive Negative Unknown Age at diagnosis of FAP, years	29 (19) 115 (76) 7 (5) 26 (13–64)
History	
Colorectal cancer Colorectal surgery	11 (7) 49 (32)
FAP classification	
Classic FAP Attenuated FAP	138 (91) 13 (9)
Ampullary adenoma	
Positive Negative	22 (15) 130 (86)
Data are presented as median (range) or n (%). FAP, familial adenomatous polyposis	

### Progression and risk factors for the progression of duodenal adenoma

After excluding 5 patients who underwent only one EGD procedure, we evaluated the remaining 146 patients who underwent two or more EGD procedures. EGD was performed approximately once per year. The median age (range) at the start and end of the observation period was 30 (16–76) and 39 (18–80) years, respectively. The median observation period (range) was 7 (0–19) years and the median number of EGD procedures (range) during the observation period was 7 (2–26).

**Fig. 2:**
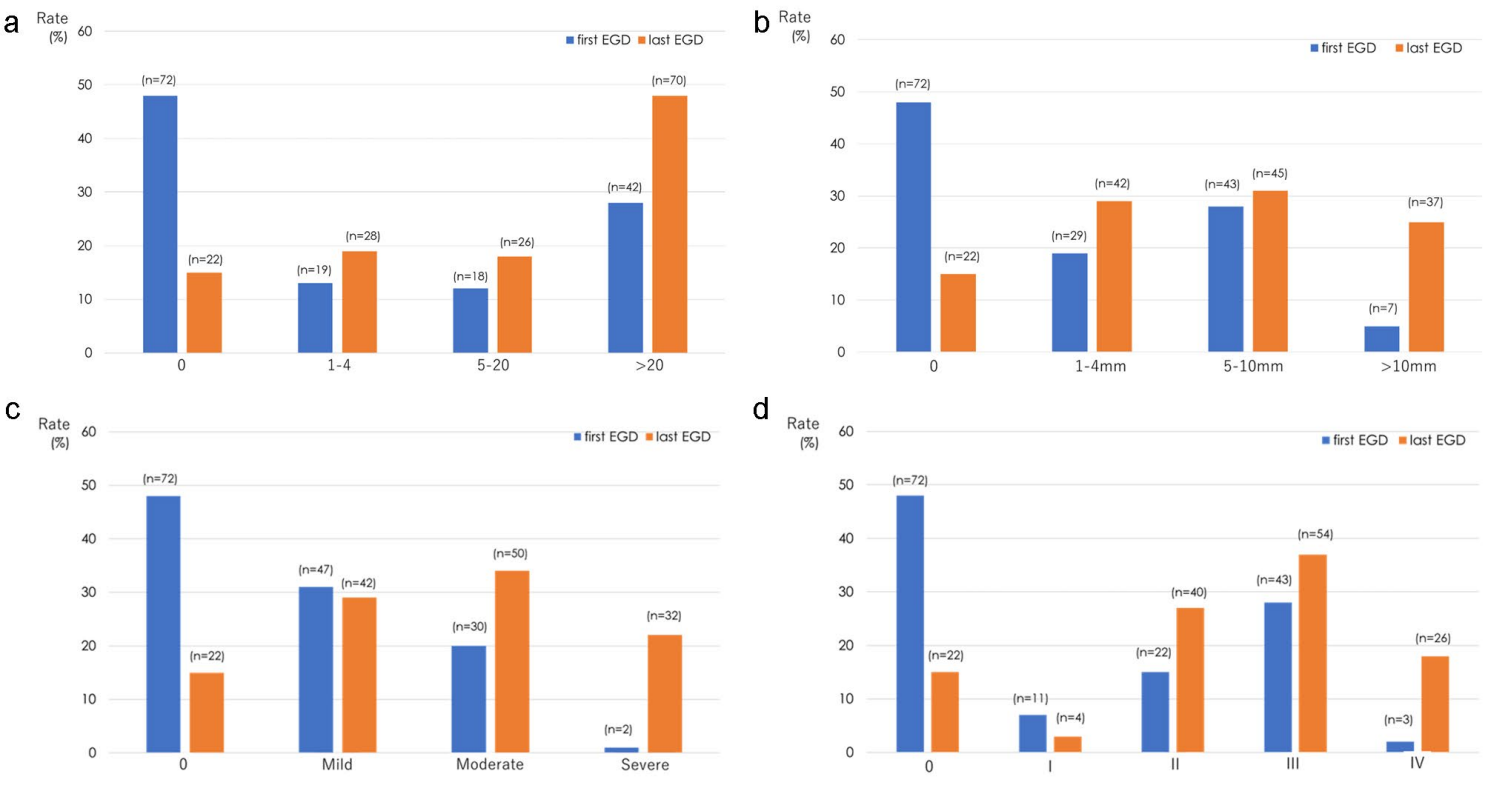
Distribution of duodenal adenomas according to Spigelman score at the first and last EGD procedures. **(a)** Number of adenomas. **(b)** Size of adenomas. **(c)** Dysplasia. **(d)** Stage. EGD: esophagogastroduodenoscopy

The prevalence of NADAs at the start of the observation period was 52% (79/151), and histologically, all were tubular adenomas. The prevalence of NADAs at the end of the observation period was 85% (124/146) and this increased 1.6-fold during the observation period. The incidence of patients with more than 20 polyps increased 1.7-fold, with polyps ≥ 10 mm increased 5-fold, with HGD increased 22-fold, and with stage IV at the last EGD increased 9-fold (Table 2; Fig. 2). 30% (21/70) of patients who did not have adenomas at the first EGD had developed at least one NADA by the last EGD. Intramucosal cancer occurred in three patients (2%), but no invasive cancer developed during the observation period because we performed endoscopic interventions for advanced adenomas before developing invasive cancer.

**Table 2:** Duodenal adenoma distribution according to Spigelman score at the first and last endoscopies

Spigelman score	First endoscopy (n = 151)	Last endoscopy (n = 146)	P-value
	n (%)	n (%)	
Spigelman score			
Number of polyps			
0 points 1 point: 1–4 2 points: 5–20 3 points: >20	72 (48) 19 (13) 18 (12) 42 (28)	22 (15) 28 (19) 26 (18) 70 (48)	< 0.001
Polyp size			
0 points 1 point: 1–4 mm 2 points: 5–10 mm 3 points: >10 mm	72 (48) 29 (19) 43 (28) 7 (5)	22 (15) 42 (29) 45 (31) 37 (25)	< 0.001
Histology			
0 points 1 point: tubular 2 points: tubulovillous 3 points: villous	72 (48) 79 (52) 0 (0) 0 (0)	22 (15) 124 (85) 0 (0) 0 (0)	< 0.001
Dysplasia			
0 points 1 point: mild 2 points: moderate 3 points: severe	72 (48) 47 (31) 30 (20) 2 (1)	22 (15) 42 (29) 50 (34) 32 (22)	< 0.001
Spigelman stage			
0 I: 1–4 points II: 5–6 points III: 7–8 points IV: 9–12 points	72 (48) 11 (7) 22 (15) 43 (28) 3 (2)	22 (15) 4 (3) 40 (27) 54 (37) 26 (18)	< 0.001

Stage progression was observed in 71% of 113 patients with stage 0 to III at the first EGD during the median observation period (2–19) of 9 years, excluding 4 patients with stage IV at the beginning and 29 patients with an observation period of ≤ 5 years without stage progression (Table 3). In addition, stage progression was recognized in 83% of 72 patients with stage 0 to I at the first EGD during the median observation period (0–19) of 10 years, excluding 9 patients with a short observation period of ≤ 5 years (Table 4). Stage progression among patients with stage 0 to III and stage 0 to I was observed significantly more often in those with classic FAP and a history of colectomy. Among patients with stage 0 to I, those with progression during the observation period were younger at the time of FAP diagnosis (Tables 3 and 4).

**Table 3:** Progression of patients with stage 0 to III (n = 113)

		Progression (n = 80)		No progression (n = 33)		P-value
**Sex**						
Male Female		34 (43) 46 (58)		12 (36) 21 (64)		0.55
**Body mass index**, kg/m²		20.7 (14.7–36.2)		21.9 (15.6–27.3)		0.80
**Alcohol intake**						
Past/current drinker Never-drinker		39 (49) 41 (51)		13 (39) 20 (61)		0.36
**Smoking**						
Past/current smoker Never-smoker		19 (24) 61 (76)		7 (21) 26 (79)		0.96
**Family history of FAP**						
Positive Negative Unknown		69 (86) 6 (8) 5 (6)		29 (88) 1 (3) 3 (9)		0.90
***Helicobacter pylori status***						
Positive Negative Unknown		12 (15) 66 (83) 2 (3)		8 (24) 25 (76) 0 (0)		0.69
**History of colorectal cancer**						
Positive Negative		6 (8) 74 (93)		1 (3) 32 (97)		0.64
**Type of polyposis**						
Classic FAP Attenuated FAP		77 (96) 3 (4)		23 (70) 10 (30)		<0.001
**Age at diagnosis of FAP**, years		24 (13–49)		27 (16–64)		0.08
**Colectomy**						
Positive Negative		33 (41) 47 (59)		5 (15) 28 (85)		0.01
**Duodenal ampullary adenoma**						
Positive Negative		14 (18) 66 (83)		4 (12) 29 (88)		0.67
**Site of*APC*** mutation (mutation at codons 279–1309)*						
Mutation within the region Mutation outside the region		41 (51) 33 (41)		20 (60) 8 (24)		0.21
**Site of*APC*** mutation*						
3’: distal mutation group from codon 1051 5’: proximal mutation group from codon 1051		23 (29) 51 (64)		14 (42) 14 (42)		0.08
**Site of*APC*** mutation*						
3’: distal mutation group (exons 10–15) 5’: proximal mutation group (exons 1–9)		44 (55) 30 (38)		21 (64) 7 (21)		0.22
**Site of*APC*** mutation (mutation at codons 1401–1580)*						
Mutation within the region Mutation outside the region		4 (5) 70 (88)		0 (0) 28 (85)		0.49
**Site of*APC*** mutation (mutation at codons 1250–1460)*						
Mutation within the region Mutation outside the region		7 (9) 67 (84)		0 (0) 28 (85)		0.21

Data are presented as n (%) or median (range)

FAP, familial adenomatous polyposis; *APC*, adenomatous polyposis coli gene

*Excluding patients with unknown mutation site

**Table 4:** Progression of patients with stage 0 to I (n = 72)

		Progression (n = 60)		No progression (n = 12)		P-value
**Sex**						
Male Female		25 (42) 35 (58)		4 (33) 8 (67)		0.83
**Body mass index**, kg/m²		20.7 (14.7–36.2)		23.1 (19.1–27.3)		0.09
**Alcohol intake**						
Past/current drinker Never-drinker		30 (50) 30 (50)		6 (50) 6 (50)		0.75
**Smoking**						
Past/current smoker Never-smoker		17 (28) 43 (72)		1 (8) 11 (92)		0.27
**Family history of FAP**						
Positive Negative Unknown		52 (87) 4 (7) 4 (7)		9 (75) 0 (0) 3 (25)		0.38
***Helicobacter pylori status***						
Positive Negative Unknown		8 (13) 50 (83) 2 (3)		4 (33) 8 (67) 0 (0)		0.45
**History of colorectal cancer**						
Positive Negative		6 (10) 54 (90)		0 (0) 12 (100)		0.57
**Type of polyposis**						
Classic FAP Attenuated FAP		58 (97) 2 (3)		3 (25) 9 (75)		< 0.001
**Age at diagnosis of FAP**, years		25.5 (13–49)		37.5 (30–64)		< 0.001
**Colectomy**						
Positive Negative		25 (42) 35 (58)		1 (8) 11 (92)		0.06
**Duodenal ampullary adenoma**						
Positive Negative		11 (18) 49 (82)		0 (0) 12 (100)		0.24
**Site of*APC*** mutation (mutation at codons 279–1309)*						
Mutation within the region Mutation outside the region		27 (45) 27 (45)		8 (67) 1 (8)		0.07
**Site of*APC*** mutation*						
3’: distal mutation group from codon 1051 5’: proximal mutation group from codon 1051		17 (28) 37 (62)		4 (7) 5 (8)		0.70
**Site of*APC*** mutation*						
3’: distal mutation group (exons 10–15) 5’: proximal mutation group (exons 1–9)		27 (45) 27 (45)		7 (58) 2 (17)		0.23
**Site of*APC*** mutation (mutation at codons 1401–1580)*						
Mutation within the region Mutation outside the region		2 (3) 52 (87)		0 (0) 9 (75)		0.66
**Site of*APC*** mutation (mutation at codons 1250–1460)*						
Mutation within the region Mutation outside the region		3 (5) 51 (85)		0 (0) 9 (75)		0.90

Data are presented as n (%) or median (range)

FAP, familial adenomatous polyposis; *APC*, adenomatous polyposis coli gene

*Excluding patients with unknown mutation site

**Fig. 3:**
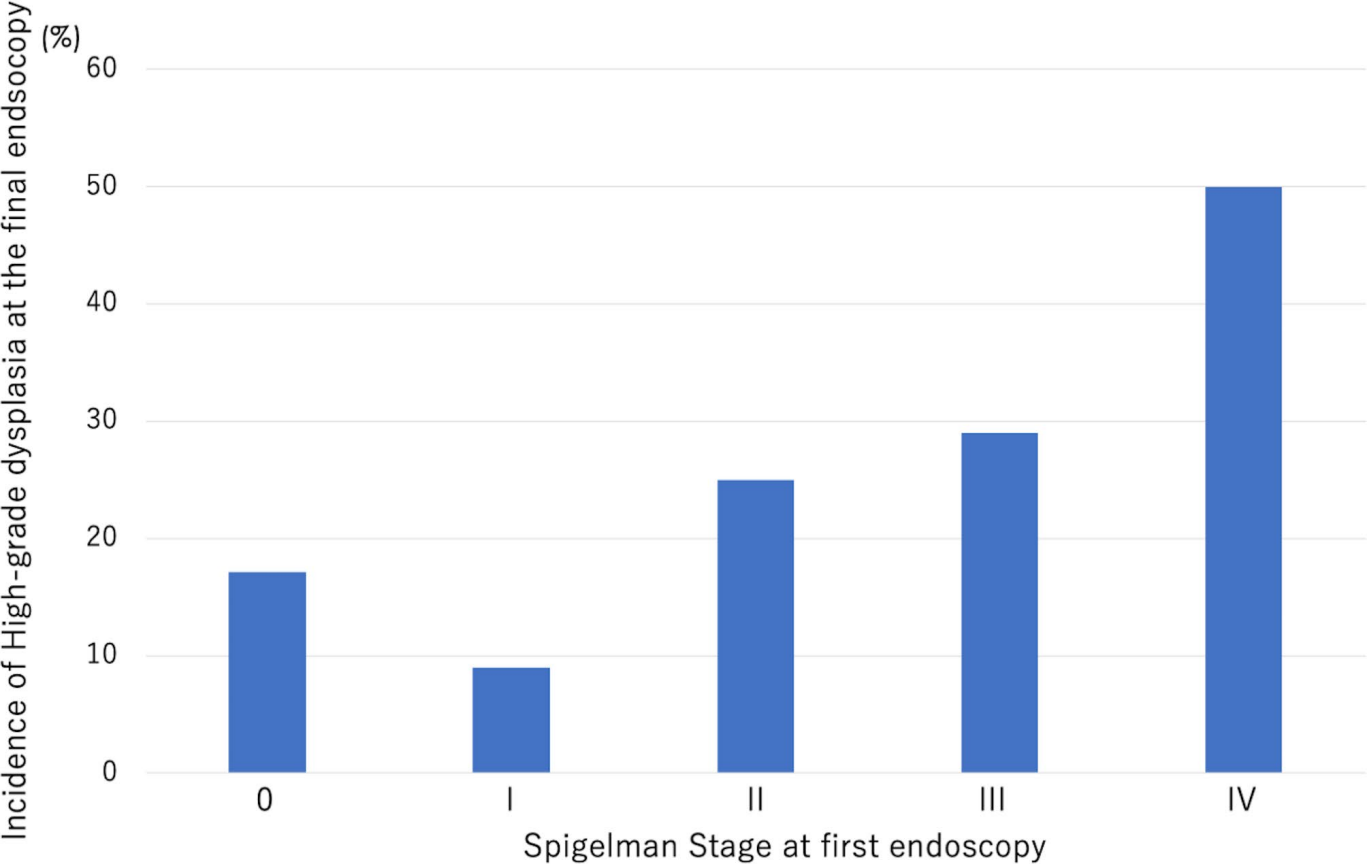
Incidence of HGD according to Spigelman stage at the first esophagogastroduodenoscopy procedure. HGD, high-grade dysplasia

HGD developed at the last EGD in 17.1% (12/70) of patients with stage 0 at the first EGD, 9% (1/11) of those with stage I, 25% (5/20) of those with stage II, 29% (12/41) of those with stage III, and 50% (2/4) of those with stage IV (Fig. 3).

### Risk factors for the lifetime development of each event until the end of the observation period

A duodenal polyp ≥ 10 mm developed in 25% of patients and was significantly more common in patients with a history of CRC, a history of colectomy, and a 3’ side mutation (Table 5). HGD occurred in 22% of patients and was significantly more common in patients with a history of colectomy (Table 6). Progression to stage IV was observed in 18% (27/146) of patients. One patient exhibited improvement from stage IV to stage III. Stage IV was significantly more common in women and patients with a history of colectomy, and it was marginally more common in those with a 3’ side mutation (Table 7).

**Table 5:** Risk factors for development of ≥ 10-mm adenoma (n = 146)

		≥ 10 mm (n = 37)		< 10 mm (n = 1069)		P-value
**Sex**						
Male Female		16 (43) 21 (57)		47 (43) 62 (57)		0.99
**Body mass index**, kg/m²		20.9 (14.7–30.1)		21.0 (15.6–37.2)		0.80
**Alcohol intake**						
Past/current drinker Never-drinker		21 (57) 16 (43)		51 (47) 58 (53)		0.29
**Smoking**						
Past/current smoker Never-smoker		8 (22) 29 (78)		28 (26) 81 (74)		0.78
**Family history of FAP**						
Positive Negative Unknown		32 (86) 1 (3) 4 (10)		94 (86) 7 (6) 8 (7)		0.87
***Helicobacter pylori status***						
Positive Negative Unknown		6 (16) 28 (76) 3 (8)		22 (20) 84 (77) 3 (3)		0.63
**History of colorectal cancer**						
Positive Negative		6 (16) 31 (84)		4 (4) 105 (96)		0.03
**Type of polyposis**						
Classic FAP Attenuated FAP		37 (100) 0 (0)		96 (88) 13 (12)		0.06
**Age at diagnosis of FAP**, years		29 (15–59)		25 (13–64)		0.22
**Colectomy**						
Positive Negative		23 (62) 14 (38)		24 (22) 85 (78)		< 0.001
**Duodenal ampullary adenoma**						
Positive Negative		6 (16) 31 (84)		15 (14) 94 (86)		0.92
**Site of*APC*** mutation (mutation at codons 279–1309)*						
Mutation within the region Mutation outside the region		26 (70) 9 (24)		53 (49) 43 (39)		0.08
**Site of*APC*** mutation*						
3’: distal mutation group from codon 1051 5’: proximal mutation group from codon 1051		15 (40) 20 (54)		32 (29) 64 (59)		0.31
Site of*APC*mutation*						
**3’: distal mutation group (exons 10–15)**		29 (78)		57 (52)		0.02
**5’: proximal mutation group (exons 1–9)**		6 (16)		39 (36)		
**Site of*APC*** mutation (mutation at codons 1401–1580)*						
Mutation within the region Mutation outside the region		2 (5) 33 (89)		4 (4) 92 (84)		0.92
**Site of*APC*** mutation (mutation at codons 1250–1460)*						
Mutation within the region Mutation outside the region		3 (8) 32 (86)		7 (6) 89 (82)		0.90

Data are presented as n (%) or median (range)

FAP, familial adenomatous polyposis; *APC*, adenomatous polyposis coli gene

*Excluding patients with unknown mutation site

**Table 6:** Risk factors for development of HGD (n = 146)

	HGD (n = 32)	No HGD (n = 114)	P-value
**Sex**			
Male Female	11 (34) 21 (66)	52 (46) 62 (54)	0.26
**Body mass index**, kg/m^2^	21.6 (14.7–30.1)	20.9 (16.8–27.5)	0.89
**Alcohol intake**			
Past/current drinker Never-drinker	15 (47) 17 (53)	57 (50) 57 (50)	0.75
**Smoking**			
Past/current smoker Never-smoker	5 (16) 27 (84)	31 (27) 83 (73)	0.27
**Family history of FAP**			
Positive Negative Unknown	30 (93) 1 (3) 1 (3)	96 (84) 7 (6) 11 (10)	0.66
***Helicobacter pylori status***			
Positive Negative Unknown	5 (16) 26 (81) 1 (3)	23 (20) 86 (75) 5 (4)	0.92
**History of colorectal cancer**			
Positive Negative	4 (12) 28 (88)	6 (5) 108 (95)	0.30
**Type of polyposis**			
Classic FAP Attenuated FAP	30 (94) 2 (6)	103 (90) 11 (10)	0.81
**Age at diagnosis of FAP**, years	26 (15–59)	26 (13–64)	0.59
**Colectomy**			
Positive Negative	17 (53) 15 (47)	30 (26) 84 (74)	0.004
**Adenoma size of ≥ 10 mm**			
Positive Negative	4 (13) 28 (88)	3 (3) 111 (97)	0.07
**Duodenal ampullary adenoma**			
Positive Negative	7 (22) 25 (78)	14 (12) 100 (88)	0.28
**Site of*APC*** mutation (mutation at codons 279–1309)*			
Mutation within the region Mutation outside the region	18 (56) 13 (41)	61 (54) 39 (34)	0.77
**Site of*APC*** mutation*			
3’: distal mutation group from codon 1051 5’: proximal mutation group from codon 1051	10 (31) 21 (66)	37 (32) 63 (55)	0.63
**Site of*APC*** mutation*			
3’: distal mutation group (exons 10–15) 5’: proximal mutation group (exons 1–9)	22 (69) 9 (28)	64 (56) 36 (32)	0.62
**Site of*APC*** mutation (mutation at codons 1401–1580)*			
Mutation within the region Mutation outside the region	2 (6) 29 (90)	4 (4) 96 (84)	0.94
**Site of*APC*** mutation (mutation at codons 1250–1460)*			
Mutation within the region Mutation outside the region	4 (13) 27 (84)	7 (6) 93 (82)	0.51

Data are presented as n (%) or median (range)

HGD, high-grade dysplasia; FAP, familial adenomatous polyposis; *APC*, adenomatous polyposis coli gene

*Excluding patients with unknown mutation site

**Table 7:** Risk factors for development of stage IV (n = 146)

	Stage IV (+) (n = 27)	Stage IV (−) (n = 119)	P-value
**Sex**			
Male Female	9 (33) 18 (67)	54 (45) 65 (55)	0.35
**Body mass index**, kg/m^2^	20.7 (14.7–30.1)	21.0 (16.8–27.5)	0.51
**Alcohol intake**			
Past/current drinker Never-drinker	13 (48) 14 (52)	59 (50) 60 (50)	0.94
**Smoking**			
Past/current smoker Never-smoker	5 (19) 22 (81)	31 (26) 88 (74)	0.57
**Family history of FAP**			
Positive Negative Unknown	24 (89) 1 (4) 2 (7)	102 (86) 7 (6) 10 (8)	0.98
***Helicobacter pylori status***			
Positive Negative Unknown	6 (22) 20 (74) 1 (4)	22 (18) 92 (77) 5 (4)	0.91
**History of colorectal cancer**			
Positive Negative	3 (11) 24 (89)	7 (6) 112 (94)	0.58
**Type of polyposis**			
Classic FAP Attenuated FAP	26 (96) 1 (4)	107 (90) 12 (10)	0.50
**Age at diagnosis of FAP**, years	25 (15–53)	26 (14–64)	0.88
**Colectomy**			
Positive Negative	14 (52) 13 (48)	33 (28) 86 (72)	0.02
**Adenoma size of ≥ 10 mm**			
Positive Negative	3 (11) 24 (89)	4 (3) 115 (97)	0.22
**Duodenal ampullary adenoma**			
Positive Negative	5 (19) 22 (81)	16 (13) 103 (87)	0.71
**Site of*APC*** mutation (mutation at codons 279–1309)*			
Mutation within the region Mutation outside the region	17 (63) 8 (30)	62 (52) 44 (37)	0.52
**Site of*APC*** mutation*			
3’: distal mutation group from codon 1051 5’: proximal mutation group from codon 1051	7 (26) 18 (67)	40 (34) 66 (55)	0.50
**Site of*APC*** mutation*			
3’: distal mutation group (exons 10–15) 5’: proximal mutation group (exons 1–9)	20 (74) 5 (19)	66 (55) 40 (34)	0.15
**Site of*APC*** mutation (mutation at codons 1401–1580)*			
Mutation within the region Mutation outside the region	1 (4) 24 (89)	5 (4) 101 (85)	0.71
**Site of*APC*** mutation (mutation at codons 1250–1460)*			
Mutation within the region Mutation outside the region	3 (11) 22 (81)	8 (7) 98 (82)	0.75

Data are presented as n (%) or median (range)

FAP, familial adenomatous polyposis; *APC*, adenomatous polyposis coli gene

*Excluding patients with unknown mutation site

## Discussion

In this study, we observed the progression of NADAs to advanced adenoma in patients with FAP. NADAs frequently become exacerbated, which may explain the need for regular surveillance and early intervention to prevent major morbidity. Studies have examined the timing and strategies of endoscopic treatment for NADAs in patients with FAP [9–11]; however, a sufficient consensus has not been achieved, and optimal management criteria have not been established. Our results provide important information regarding the management of NADAs in patients with FAP.

The reported prevalence of NADAs in patients with FAP ranges from 30 to 90% but increases after the age of 40 years [24, 25], and the SS also worsens with age [10, 12, 26, 27]. In the present study, the prevalence of NADAs at the start of the observation period was 52%. However, the numbers of patients with NADAs, more than 20 polyps, polyps > 10 mm, HGD, and stage IV at the final EGD were substantially increased during the median 7-year observation period. Although 15% of patients did not develop NADAs during the observation period, the risk of developing NADAs increased over time, as previously reported, and even patients with early-stage NADAs showed frequent progression. Stage progression was observed in 71% of patients, and the same tendency was observed in patients with stage 0 to I at the first EGD. Because the incidence of HGD at the last EGD was higher among patients with a more advanced stage at the first EGD, it is desirable to prevent stage progression. Furthermore, the incidence of HGD in patients with stage 0 at the first EGD was 17% at the last EGD. Therefore, it might be better to consider surveillance EGD for all patients with FAP.

No patients in the present study developed invasive cancer during the study period because we recommended treatment intervention before the development of invasive cancer. Groves et al. [2] reported that SS IV was a risk factor for developing duodenal cancer at the time of the first endoscopy, and was a risk factor in patients who developed duodenal cancer. In other studies, 63% of patients with stage IV disease at the time of the first EGD developed HGD [10, 28], and a ≥ 10-mm adenoma was shown to be a risk factor for HGD [6]. Therefore, advanced duodenal adenomas and SS IV have been cited as the main risk factors for the development of duodenal cancer, and can be considered surrogate measurement indices for the development of invasive cancer in this study. Although the usefulness of SS is controversial, there is currently no alternative measurement [20]. In addition, Thiruvengadam et al. reported that polyp size and HGD were associated with cancer development [20]. Therefore, our assessment using advanced adenoma and SS simultaneously is reasonable.

In the present study, we found no correlation between the development of severe duodenal adenomatosis or duodenal cancer and the specific location of the mutation in *APC*, although the risk factors for advanced duodenal adenoma and SS IV were classic FAP, a history of CRC, a history of colectomy, and a 3’ side mutation. Therefore, it might be better to perform more intensive surveillance for patients with these risk factors instead. In the current study, no patients developed invasive cancer with annual surveillance; thus, annual surveillance may be sufficient, even for high-risk patients. However, for low-risk patients such as those with attenuated FAP and small polyps, the interval of surveillance endoscopy can be prolonged.

In the current study, 18% of the patients progressed to stage IV. Surgical intervention should be considered for such patients because of the risk of the development of duodenal cancer. Because surgical intervention (typically pancreatoduodenectomy) for the treatment of duodenal cancer or stage IV disease can be extremely invasive and is accompanied by severe sequelae, it should be avoided if possible. This is especially true for patients who have undergone total colectomy, which is a standard intervention for classic FAP. Although endoscopic intervention has not been actively recommended because of the high risk of adverse events and frequent recurrences, we have adopted safer endoscopic interventions for the management of duodenal neoplasms in patients with FAP [29]. We also recommended treatment intervention for patients with an advanced duodenal adenoma in the present study, and no invasive cancer developed in our cohort. This indicates that early intensive endoscopic management can avoid development of duodenal cancer or progression to SS IV in patients with FAP [10, 29–31].

Three main limitations of this study should be considered. First, this was a retrospective study involving a small number of patients in a small number of facilities. Therefore, the study findings may not be highly generalizable. However, we enrolled all consecutive patients who met the inclusion criteria, and the ability to collect detailed, high-quality endoscopic data from two linked facilities was advantageous. In addition, we focused on patients who met the diagnostic criteria for FAP and were positive for the *APC* mutation; therefore, we were able to extract and examine more reliable data. In future studies, we hope that patient data will be collected at multiple facilities using registries. In addition, we hope our data will contribute to a future meta-analysis, which can provide more robust information. Second, the observation period was too short to determine the real lifetime risk of duodenal neoplasms. This might have caused an underestimation of the risk of duodenal neoplasms; therefore, the study findings should be interpreted as at least a progression of duodenal manifestations in most of the patients with FAP. Finally, our study did not include ampullary adenoma to clarify the progression of NADAs separate from ampullary adenoma. Because half of duodenal cancers were reported to develop from ampullary adenomas, we should consider them as well as NADAs for the long-term prognosis of patients with FAP. However, the treatment strategies for ampullary adenoma and NADAs are different, and we needed to investigate them separately.

## Conclusions

NADAs in patients with FAP frequently become exacerbated, and all patients with FAP should be surveyed to prevent the development of duodenal cancer. An early, minimally invasive therapeutic intervention should be considered to avoid disease progression.

## Data Availability

The following data will be made available with publication: complete deidentified patient data set (contact Yoji Takeuchi; e-mail, yoji.endoscopy@oici.jp). The following supporting documents will be made available with publication: analytic/statistical code (contact Yoji Takeuchi; e-mail, yoji.endoscopy@oici.jp). These data will be made available for any purpose to researchers whose proposed use of the data has been approved.
